# Evaluation of Antibiotic-Based Selection Methods for *Camelina sativa* Stable Transformants

**DOI:** 10.3390/cells11071068

**Published:** 2022-03-22

**Authors:** Abraham Ontiveros-Cisneros, Oliver Moss, Alex Van Moerkercke, Olivier Van Aken

**Affiliations:** Molecular Cell Biology, Department of Biology, Lund University, Sölvegatan 35, 223 62 Lund, Sweden; abraham.ontiveros_cisneros@biol.lu.se (A.O.-C.); oliver.moss@slu.se (O.M.)

**Keywords:** Camelina, antibiotic selection, camelina crossing, qRT-PCR, BASTA, hygromycin

## Abstract

*Camelina sativa* (Camelina) is an oilseed crop that in recent years has gained importance due to its closeness to the plant model organism *Arabidopsis thaliana* (Arabidopsis), its low agronomical requirements, and the ability to grow under temperate conditions. To explore all the agronomical and biotechnological possibilities of this crop, it is important to evaluate the usability of the molecular procedures currently available for plants. One of the main tools for plant genetic modification and genetic studies is stable plant transformation. In the case of Arabidopsis, as well as Camelina, floral dipping is the easiest and most used method, which is followed by a selection for stable transformants. Commonly used selection methods for Camelina involve *Discosoma* sp. red protein (DsRed) fluorescence screening. However, many widely used plant transformation vector systems, for example those used in Arabidopsis and grasses, rely on antibiotic resistance selection. In this study, we evaluated the usability of different antibiotics including kanamycin (Kan), hygromycin (Hyg) and BASTA, and propose optimised protocols for selecting T1 and subsequent generation Camelina transformants, as well as crossing of Camelina lines expressing different transgenes. Finally, we also showed that overexpression of genes encoding enzymes from the seco-iridoid pathway of *Catharanthus roseus* using Hyg or BASTA-based expression constructs could be successfully achieved in Camelina, demonstrating the potential of these methods for metabolic engineering. Overall, in this study we show an efficient way to sterilize seeds, handle and perform selection of Camelina for use with transformation vectors designed for *Arabidopsis thaliana*. We also demonstrate a successful method to cross *Camelina sativa* and provide qRT-PCR results to prove its effectiveness.

## 1. Introduction

*Camelina sativa* (Camelina), an oilseed plant adapted to temperate growing regions, is regarded as a low-input crop resistant to common diseases and pests of the *Brassicaceae* family [1]. Camelina oil has different uses and can be produced for industry, food, and pharmaceutical purposes [2]. Due to its production of triacylglycerol (TAGs), its most important use is the production of biofuel; however, it has not benefited significantly from modern breeding or transgenic approaches, which could enhance or change the type of TAGs produced [3,4]. Nowadays, the production of this crop is very promising for temperate regions (e.g., Central and Northern Europe) for its adaptability, low requirements, and short crop cycle [1]. Regarding its yield, according to several trials, it can reach over 3 tonnes/ha, where a positive correlation between seed yield and oil content was found [5]. Compared to a crop such as oilseed rape, Camelina has a similar oil yield, but it has lower pesticide and fertiliser requirements [6].

Camelina, a self-pollinating crop with an allohexaploid genome (2n = 6x = 40), is related to the model plant *Arabidopsis thaliana* (Arabidopsis) [7], from which abundant tools and knowledge can be translated into Camelina for food and non-food applications. Considering the number of methods available for Arabidopsis and the economic importance of Camelina as a sustainable crop for oil production, it is important to explore the application of different molecular tools in this emerging crop. Genetic transformation is one of the fundamental procedures for performing metabolic improvement as well as breeding improved varieties of any crop. Since Camelina is gaining relevance for the scientific and agricultural community, it is important to report the transfer of known procedures of genetic transformation methods from Arabidopsis to Camelina. 

Genetic transformation is followed by positive selection or screening. In the case of *Brassica napus*, another oilseed crop, the selection occurs using a red fluorescence gene (DsRed) in the vectors used to transform hypocotyls [8]. In the case of soybean, *Agrobacterium tumefaciens*-mediated transformation is followed by selection using hygromycin [9]. Other transformation methods reported for oilseed crops (Canola) include microspore transformation, particle bombardment, and epicotyl and internodes transformation followed by selection using spectinomycin [10]. 

On the other hand, one of the most commonly used methods to insert a transgene into *Arabidopsis thaliana* is floral dipping, and positive transformants can then be selected using the corresponding antibiotic resistance. With some adaptations, floral dipping is a method that is also widely used in *Camelina sativa* transformation [11]. Interestingly, another study showed that Camelina can be transformed in a similar way as Arabidopsis, but for selection purposes the seeds are sterilized in liquid, which could complicate handling [3]. Most of the literature reports selection methods based on DsRed fluorescence or BASTA spraying on soil, which requires high doses of the antibiotic [11,12]. Other studies show the use of markers such as chlorsulfuron, which is not a very common resistance in plasmids available for plant transformation, or even marker-free selection using high-throughput filter paper-based PCR screening [3]. On the other hand, many commonly available plasmids for stable transformation of plants use antibiotic-based selection with hygromycin, kanamycin or BASTA [13,14]. It would therefore be useful to have the possibility to directly use such plasmids for Camelina-related research and applications. In vitro selection of transformants is particularly useful and potentially more economical for selection of single T-DNA locus and homozygous lines compared with, for example, soil-based selection. Having a range of workable selection markers is also useful when crossing or super-transforming lines to combine multiple traits, for instance for metabolic engineering of complex pathways. Here we present an evaluation of different antibiotics and doses for in vitro selection of *Camelina sativa* primary transformants from dipped seeds, as well as for later generations for segregation analysis.

## 2. Materials and Methods

### 2.1. Plant Material and Growth Conditions

*Camelina sativa* wild type (WT) was used for all the transformation and selection experiments. For floral dipping transformation, five seeds were sown per 10 cm pot with draining holes filled with soil mix with vermiculite and perlite at a 4:1:1 ratio. Three plants per pot were retained and the extra seedlings discarded. The Camelina trays were placed in a greenhouse where the growth conditions were 16 h light/8 h dark, approximately 400–600 μmol photons m^−2^ s^−1^ and 20/16 °C (day/night) and the humidity maintained at 60%. 

To determine the right dose for transformant selection, WT Camelina seeds were plated on different antibiotics at different concentrations. Along with hygromycin and BASTA, kanamycin was also included in the evaluation. Initially, Murashige and Skoog (MS) media plates were prepared with no antibiotic, kanamycin at 25, 50 and 100 mg/L, hygromycin at 7.5, 15 and 22.5 mg/L and BASTA at 2.5, 5 and 7.5 mg/L. For each of the three antibiotics, the doses corresponded to half, full and one and a half of the standard doses for *Arabidopsis thaliana* on plate selection [15,16]. Subsequently, double and triple dose were tested for Kanamycin and BASTA. 

Prior to plating, seeds were sterilized by washing with 20% bleach (12% NaClO) three times for 7 min and rinsing with MQ water three times under the laminar flow hood. A total of 50 seeds were transferred to each plate and left for 48 h at 4 °C for stratification. After stratification, plates were grown for 20 days in standard long day plant growth conditions (16 h light/8 h dark, approximately 120 μmol photons m^−2^ s^−1^). 

For T1 pB2GW7 transformants, a tray full of soil mix was prepared where all the seeds from 12 dipped plants were sown, placed for stratification for 48 h at 4 °C and then set to grow under greenhouse conditions. Seedlings at the two-leaf stage were sprayed three times during a 2-week span (with a 3-day rest interval between sprays) with a solution of BASTA 0.01% (*w*/*v*) in water and Silwet-77 (500 μL/L). This method was preferred over plate selection due to the high number of seeds required for transformant screening. 

In the case of pH2GW7 transformants, MS media plates were prepared with 22.5 mg/L hygromycin. Seeds were sterilized prior to plating by using a chlorine gas sterilisation method with a solution of 40 mL MQ water, 6.25 mL bleach and 1.75 mL HCl. The seeds were incubated overnight inside a desiccator. For T2 generations and forward, plate selection was used to evaluate the homozygosity of the Camelina lines and to avoid high doses of BASTA.

Camelina crosses were generated by emasculating pH2GW7 positive transformants and pollinating them with mature and pollen-producing flowers from pB2GW7 positive transformants. The emasculation was conducted on early flower buds by removing the surroundings of the style with tweezers under a microscope. The buds which were not pollinated were removed from the stem. The resulting seeds were collected and sown in soil trays. To ensure successful crossing, BASTA selection on soil was done as previously described and the surviving plants were transferred to new soil.

### 2.2. Plasmid Cloning and Transformation

Three different binary Gateway plasmids were used in this study [13]. To test BASTA as a selection marker in Camelina after floral dipping, the plasmid pB2GW7, which carries the *G8O* gene from *Catharanthus roseus* driven by a 35S promoter, was used. Similarly, to test Kanamycin we used pK7GW2, which carries *G80* driven by a 35S promoter. The third construct, which was used for testing hygromycin, had a pH2GW7 backbone carrying a *GES* gene from *Catharanthus roseus* driven by a 35S promoter. The plasmids were introduced to the chemically competent *Agrobacterium tumefaciens* strain GV3101 by the heat-shock method. The selection of transformed bacteria was performed on LB plates containing 100 μg/mL spectinomycin, 100 μg/mL rifampicin and 40 μg/mL gentamycin. The presence of the correct constructs was confirmed by colony PCR. 

Agrobacterium growth and Camelina dipping were carried out in the same way as described previously in the literature [11]. Dipped plants were left in the shade covered in plastic for 24 h. After transformation, plants were left for growing and drying under the greenhouse conditions previously described. 

### 2.3. G8O and GES qRT-PCR

RNA was extracted from positive transformants and crosses surviving antibiotic selection using a Spectrum^TM^ Plant Total RNA Kit from Sigma. The RNA samples were normalised at 100 ng/μL and cDNA was synthetized using a Bio-Rad iScript Reverse Transcription Kit using 500 ng of RNA per sample. The RT program was as follows: 5 min at 25 °C, 25 min at 42 °C and 1 min at 95 °C. The samples were diluted 7:1 with MQ water. For qRT-PCR, primers pairs were designed to bind *G8O* and *GES*, while *ACTIN2* (*ACT2*) from Camelina was used as a housekeeping gene (Table 1). The qRT-PCR mix was performed with 2.5 μL Sso Advanced^TM^ Universal SYBR Green Supermix, 0.25 μL primers (10 μM) and 0.25 μL H_2_O, along with 2 μL of diluted cDNA. The qRT-PCR program was performed in a CFX384 Touch Real-Time System following previously described methods [17]. 

## 3. Results

### 3.1. Antibiotic Concentration Evaluation on Wild Type Camelina

**Figure 1:**
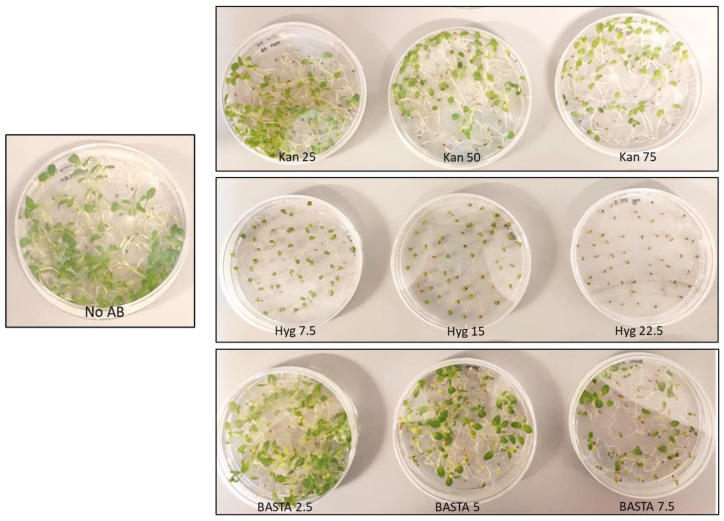
Dosage experiment for kanamycin (Kan), hygromycin (Hyg) and BASTA in WT Camelina seeds after 20 days of growth. Doses in mg/L. No AB: MS media without added antibiotics as a control.

**Figure 2:**
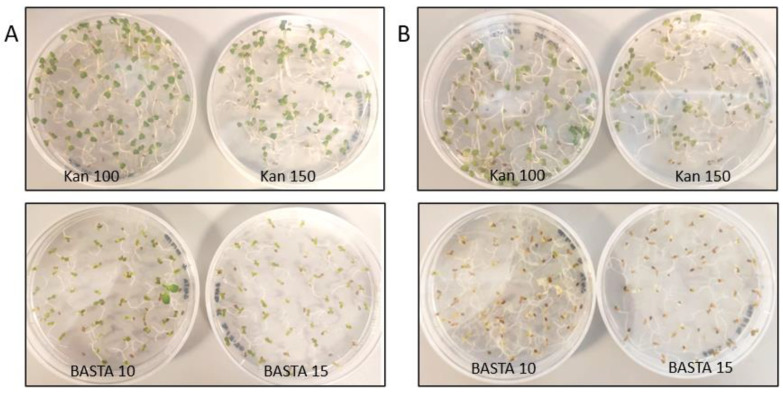
Kanamycin (Kan) and BASTA selection at higher doses. Selection of transgenic Camelina grown in vitro on MS media plates containing variable concentrations of antibiotic. Doses are expressed in mg/L. Picture taken after (**A**) 20 days growth, or (**B**) 30 days growth.

As a first step in evaluating selection methods for transformed Camelina using antibiotics, we established a suitable range of antibiotic concentrations to eliminate wild type Camelina. For kanamycin and BASTA, we started with doses corresponding to 0.5–1.5 times the standard concentration used for Arabidopsis selection (50 mg/L for kanamycin and 5 mg/L for BASTA). After growing Camelina WT for 20 days on MS plates (Figure 1), we observed no obvious difference compared with the no-antibiotic plate for any of the tested kanamycin or BASTA doses. We thus increased the kanamycin dose to 100–150 mg/L, and to 10–15 mg/L in the case of BASTA. After 20 days, both antibiotics still showed green surviving plants (Figure 2), but in the case of BASTA we could observe a smaller plant size compared to control conditions. Some plants could still largely escape selection at 10 mg/L Basta, but this was not observed at 15 mg/L. Plates were left ten more days to further evaluate the selection. In the case of BASTA, both doses showed dead plants after a total of 30 days, whereas the kanamycin plates still showed surviving plants, although they were a paler green color. 

In the case of hygromycin, an effective selection against wild type plants was observed at 20 days of growth at a dose of 22.5 mg/L, corresponding to 1.5 times the standard Arabidopsis dose. Though the wild type Camelina plants clearly suffered and were much smaller on 7.5–15 mg/L of hygromycin compared with control plates, they did remain viable and green. In conclusion, suitable concentrations for hygromycin and BASTA selection against wild type Camelina were found, which were approx. 1.5 times higher than standard doses used for Arabidopsis selection. However, Camelina appeared to be very resistant to high doses of Kanamycin, even at three times the standard Arabidopsis dose.

### 3.2. Camelina Transformant Selection

**Figure 3:**
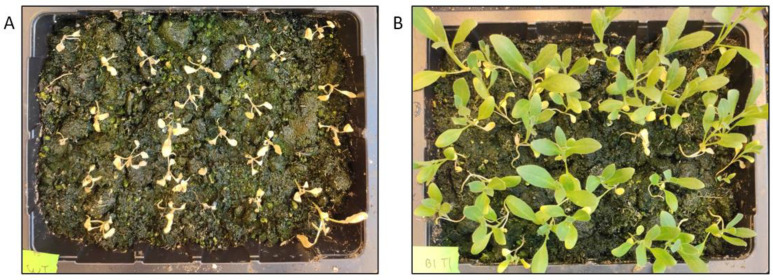
BASTA on-soil selection of T2 transformants. Soil-grown transformants were selected by spraying with 100 mg/mL BASTA starting from the two-leaf stage. The picture was taken after spraying three times in total, approximately two weeks after the first dose. (**A**) *WT Camelina sativa* (**B**) Segregating pB2GW7-G8O transformants.

Next, we wanted to test the optimal conditions for selecting transgenic Camelina transformed with Hyg, BASTA and Kan resistance markers. As spraying soil-grown plants with BASTA was previously reported as a selection method [12], we tested wild type and segregating T2 populations transformed with a BASTA transgene. Plants were sprayed with a 100 mg/L BASTA solution for the first time after they reached the two-leaf stage (approx. one week old), followed by two more rounds of spraying approx. every three days (Figure 3). The on-soil selection by spraying a 100 mg/L BASTA solution appeared to be an effective method to eliminate all WT Camelina and select positive transformants. We also tried the BASTA spraying method on densely sown trays to select T1 primary transformants, which was also successful and made the transgenic plants clearly identifiable. 

**Figure 4:**
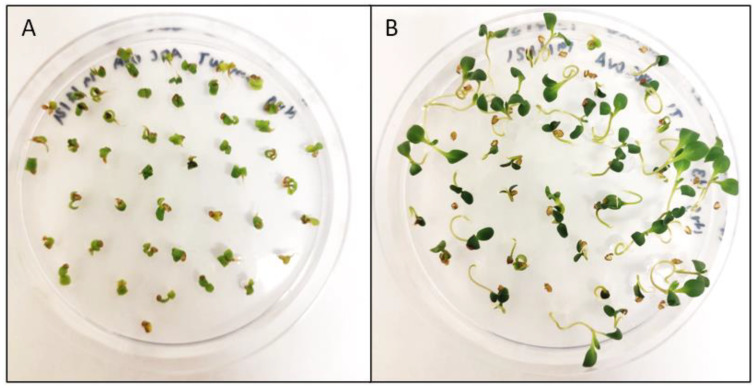
Selection of transgenic Camelina on MS media containing 22.5 mg/L Hygromycin. Pictures were taken after 14 days. (**A**) WT *Camelina sativa* (**B**) Segregating T2 generation of pH2GW7 transformants (hygromycin-resistant).

**Figure 5:**
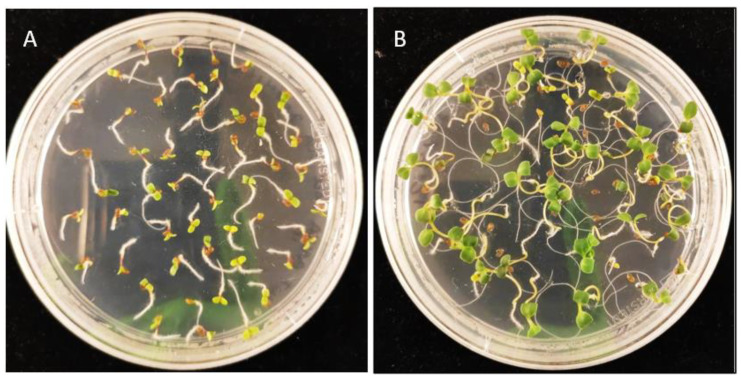
Selection of transgenic Camelina on MS media containing 15 mg/L of BASTA. Pictures taken after 14 days. (**A**) WT *Camelina sativa* (**B**) Segregating T2 generation of pB2GW7 transformants (BASTA-resistant).

In the case of in vitro selection, both hygromycin at 22.5 mg/L and BASTA at 15 mg/L showed a clear difference between WT and segregating positive transformant populations after 14 days of growth, as can be observed in Figure 4 and Figure 5. Screening large quantities of floral dipped T1 seeds was also tested on MS plates with Hyg or BASTA. Due to the large number of seeds, the sterilisation method was important to obtaining optimal results. We found that liquid sterilisation by washing with bleach solutions was effective to sterilize small seed batches of around 50–100 seeds but resulted in too much contamination when sterilising in bulk for primary transformant selection. Instead, dry sterilisation with chlorine gas was a more effective way to simultaneously sterilise larger seed batches. It was important to maximise the spreading of the seeds across a larger surface area to allow sufficient penetration of the chlorine gas between the seeds. The optimised concentrations of Hyg at 22.5 mg/L and BASTA at 15 mg/L were successful in identifying primary transformants among T1 dipped seeds.

**Figure 6:**
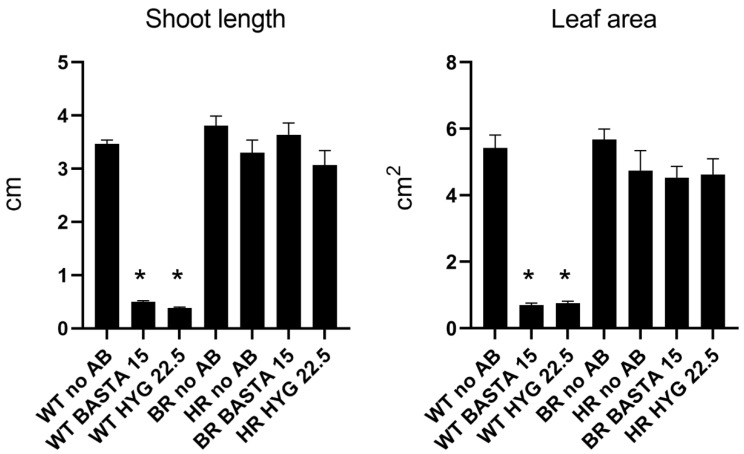
Antibiotic effects on shoot length and leaf area. For both graphs the first three columns correspond to WT Camelina grown in vitro with no antibiotics, BASTA 15 mg/L or Hygromycin 22.5/mg, respectively. Next, BR (BASTA-resistant) and HR (Hyg-resistant) plants grown with no antibiotics, followed by BR and HR lines grown on the antibiotic they carry resistance for. Measurements were performed using 14-day-old seedlings. Error bars indicate Standard Error. Statistical significance was calculated using Student’s *t*-test comparing each treatment with WT no AB. Only treatments with (*) showed significant difference (*p*-value < 0.05).

Consequently, we tested the effect of both antibiotics at the doses we found to be effective for Camelina: Hyg 22.5 mg/L and BASTA 15 mg/L. WT Camelina, Hyg-resistant and BASTA-resistant plants were grown in Petri dishes with no antibiotics, Hyg 22.5 mg/L or BASTA 15 mg/L. After 14 days of growth, various parameters such as leaf area and shoot length were measured (Figure 6). Overall, it was observed that all the seeds germinated under all conditions, and they developed true leaves, except for WT Camelina grown in either of the antibiotics. For both leaf area and shoot length, only WT Camelina exposed to BASTA and Hygromycin showed significantly reduced growth when compared with WT Camelina growing without antibiotics. The selected concentrations of antibiotic thus allowed plants containing the resistance genes to grow healthily, while wild type plants were effectively selected against.

### 3.3. Selection of Transgenic Lines Overexpressing Seco-Iridoid Pathway Enzymes from Catharanthus roseus

As genetic transformation is an important tool for metabolic engineering in crops, we transformed wild type Camelina with CaMV35S-based overexpression constructs. We chose two enzymes that are among the first steps of the (seco)-iridoid pathway from *Catharanthus roseus* leading to production of monoterpenoid indole alkaloids (MIAs) [18]. Several MIAs have valuable properties such as the high-value anti-cancer drugs vinblastine and vincristine, whereas other MIAs such as iridoids can act as insect repellents [19]. We cloned *geraniol synthase* (*GES*), which converts geranyl diphosphate into geraniol, from *Catharanthus roseus* into pH2GW7 for Hyg selection. We also cloned the next step in the pathway *geraniol 10-hydroxylase/8-oxidase* (*G8O*), which converts geraniol into 8-hydroxygeraniol, from *Catharanthus roseus* into the pB2GW7 vector and transformed it into Camelina by floral dipping. The T1 primary transformants were selected by spraying of soil-grown plants with 100 mg/L BASTA for pB2GW7, or in vitro on MS plates with 22.5 mg/L Hyg for pH2GW7, respectively. 

**Figure 7:**
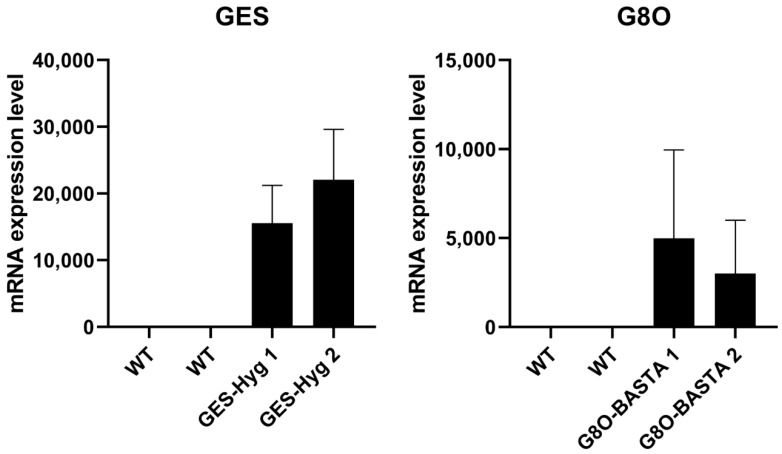
*GES* and *G8O* relative mRNA expression to WT in T1 Camelina transformants. *GES* and *G80* are transgenes originating from *Catharanthus roseus*, while *ACT2* is an actin gene from Camelina. Due to the lack of the transgene in WT, samples were normalised to WT. In both graphs two WT samples were used along with 2 positive transformants. The error bars represent standard deviation (SD) between samples. GES-Hyg 1 and GES-Hyg 2 are two plants which carry the *GES* gene along with a hygromycin resistance gene from the pH2GW7 backbone. G8O-BASTA 1 and G8O-BASTA 2 are two plants which carry the G8O gene along with a BASTA resistance gene from the pB2GW7 backbone.

Basta- or Hyg-resistant plants were subsequently tested for transgene expression by qRT-PCR. The house keeping gene *ACT2* showed a consistent expression in WT Camelina as well as for positive transformants, with CT values around 22 to 23 cycles. As the selected *GES* and *G8O* are not endogenously expressed in Camelina, the wild type plants showed no specific amplification of the qRT-PCR products, so the CT value was set to 40 and their expression fold considered as 1× (Figure 7). In contrast, the selected Hyg-resistant plants showed high relative transcript abundance for *GES*, with CT values around 25 cycles, while Basta-resistant plants showed high transcript levels for *G8O*, with CT values around 28 cycles (Figure 7). We also tried floral dipping followed by Kan-based selection using the pK7WG2 vector (containing *G8O*). However, as observed above, no clear counter-selection of wild type plants could be achieved, and no clear Kan resistant T1 plants could be identified. In summary, these findings show that overexpression of transgenes can be achieved in Camelina using Hyg and BASTA-based selection. 

### 3.4. Selection of F1 Lines Overexpressing Both GES and G8O Seco-Iridoid Pathway Enzymes by Crossing of Camelina

**Figure 8:**
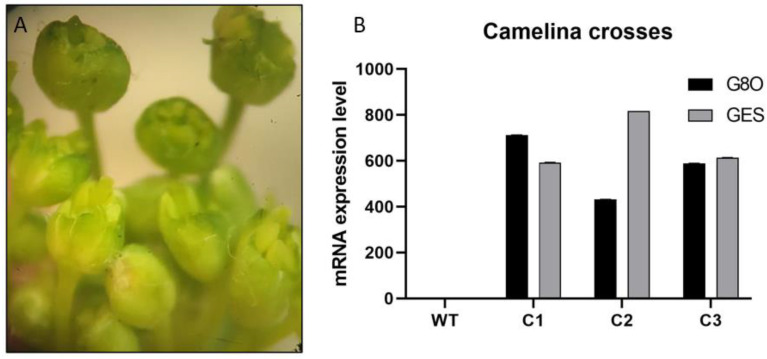
(**A**) Emasculated Camelina flower buds used as the female part in crossing. (**B**) *GES* and *G80* relative mRNA expression to WT in F1 Camelina crosses using *ACT2* as a house keeping gene. *GES* and *G80* are transgenes originating from *Catharanthus roseus*, inherited from female and male parental plants respectively, while *ACT2* is an actin gene from Camelina. C1, C2 and C3 are three of the 15 crosses that survived BASTA selection.

Genetic engineering of metabolic pathways often involves the introduction of multiple genes to build a complete pathway. Furthermore, engineering of multiple pathways which have at least partially common enzymatic steps could be desired. Therefore, it can be useful to genetically combine transgenes expressed in different lines by crossing the parental lines. Since BASTA selection is the easiest in Camelina, we attempted to produce Camelina lines carrying both GES and G8O enzymes by crossing GES Hyg-resistant plants as the female, with pollen from G8O BASTA-resistant plants. We could not find clear protocols for crossing Camelina in the literature, so we initially attempted crossing Camelina with a similar method we normally use to cross Arabidopsis (by pollinating flowers that were not opened, but from which the stigma was just emerging from the closed sepals/petals, with pollen from a mature and fully opened donor flower). This crossing method was performed several times but was, however, not successful, with no seeds coming from the crossed female flower surviving BASTA selection. Therefore, we tried a different strategy: emasculating the acceptor flower buds leaving the unpollinated style exposed and undamaged (Figure 8A). This emasculation was performed using fine sharp tweezers under a dissection microscope. The emasculated buds were pollinated with pollen from mature BASTA resistant flowers, and the surrounding unpollinated buds were removed from the acceptor plant. From a total of 40 buds, 20 continued developing healthily and produced around five to eight seeds per bud, after being emasculated and pollinated (Figure 8A). All the seeds were sown in soil and later sprayed with 100 mg/L BASTA, which is the resistance gene inherited from the male parental side, to verify the crossing was successful. Fifteen plants survived the BASTA selection and were then transferred to new pots. These plants were subsequently tested for expression of both transgenes by qRT-PCR. As the selected *GES* and *G8O* are not endogenously expressed in Camelina, the wild type plants showed no specific amplification of the qRT-PCR products. However, the transcript of both transgenes could be detected in all tested plants, with most of the F1 plants showing high relative transcript abundance for both GES and G8O (Figure 8B).

## 4. Discussion and Conclusions

This work showed that with some optimisation the well-known *Arabidopsis thaliana* antibiotic-based selection methods, doses, seed sterilisation and plating can be used in Camelina. BASTA at 15 mg/L and Hygromycin at 22.5 mg/L seem to be the optimal doses for Camelina primary transformant selection on MS plates after 20 days. However, for T2 generation and forward, 14 days seem sufficient to obtain a clear difference between WT and positive transformants. In the case of Kanamycin, contrary to previous studies [6], at least in our hands, even high doses and long growth times did not work effectively in discriminating potentially positive transformants from WT. Therefore, we recommend using BASTA for its easy spray-based selection of primary transformants in soil, without the potential risks of contamination when using in vitro selection. In addition, Hyg can be used successfully for in vitro T1 selection as demonstrated here, though care should be taken to thoroughly sterilise the seeds. For handling large numbers of seeds, it is thus recommended either to use BASTA spraying on soil or prepare plates as suggested in this study. Liquid sterilisation is not recommended for this purpose, since Camelina seeds retain a large amount of water and tend to clump. Gas sterilisation seems to be the simplest method to sterilise a large number of seeds and also makes handling easier for plating. This gas sterilisation is especially recommended for T1 when many seeds are required. When sterilising, it is recommended to spread the seeds across a large surface area to allow adequate exposure to the chlorine gas. When plating, it is easier to use sterile tweezers than to handle the seeds with a pipette for both liquid and gas sterilisation. From T2 and forward, both sterilisation methods are reliable since segregation screening can be performed with a lower number of seeds (~50). Gas sterilisation is the easier method for seed handling, also when many small batches of seeds need to be screened (e.g., for selecting homozygous T3 lines). If overnight incubation is not desired, liquid sterilisation is an effective alternative, though one must be careful not to extend the bleach incubation times for too long when processing many separate tubes. Step-by-step procedures of the selection methods described in this manuscript are provided in the Supplementary Information File S1.

All the antibiotic-based selection methods evaluated here were validated not only phenotypically, but also by analysing the gene expression of the transformed plants for the transgene. Additionally, we described a method to successfully cross Camelina lines, which could be used to engineer, for example, more complex metabolic pathways. We suggest to use the combination of Hyg and BASTA for Camelina crossing, with the BASTA line as pollen donor. Furthermore, this procedure was validated by checking the relative expression of both G8O and GES genes by qRT-PCR in the crossed progeny.

Reported methods for similar oilseed crops include screening for DsRed fluorescence, which requires special equipment [8] or laborious preparation of samples for transformation [10]. Even for Camelina itself, the reported methods include the use of DsRed or the spraying of high amounts of BASTA [11,12]. Selection using chlorsulfuron has also been reported for Camelina, but uses the acetolactate synthase (*ALS*) selectable marker gene, which is relatively uncommon in standard plant transformation plasmids [3]. Compared with these transformation methods, this study demonstrates the use of a reliable and simple transformation method such as floral dipping [11] followed by straight-forward and efficient selection, which does not require special equipment or high amounts of antibiotics. The required seed sterilisation for sowing on plates is also uncomplicated and makes handling simpler as compared with other protocols using liquid sterilisation [3]. 

Although we could not successfully use Kan as a marker for selection of floral dipped Camelina, Kan has been previously reported for selecting Camelina positive transformants [20]. In that study, transformation was performed in apical meristems followed by tissue culture and root induction. The author also defined an optimal dose for kanamycin but again using micro-propagated cells [21]. Thus, it could be possible that Camelina is only susceptible to kanamycin in tissue culture, but not when grown from seed. This seedling regenerating method also uses GFP selection, which is a widely used marker in selection of positive transformants and is a recurrent element on plasmids available for plant transformation [14,22]. These methods show advantages such as higher transformation efficiency, in the case of apical meristems [20] and the use of a well-known marker such as GFP. However, contrary to the selection strategy we present here, these methods require special equipment and delicate handling of the transformed plants and sterile cultures. 

In conclusion, we have evaluated the usability of commonly used plant selectable markers for selection of transgenic Camelina and developed useful procedures. It is relevant to report on the effectiveness of markers such as hygromycin and kanamycin, since they are not commonly used for *Brassicaceae* (apart from Arabidopsis) positive transformants selection [23]. We were able to use these methods to stably express genes encoding enzymes of the *Catharanthus roseus* seco-irodoid pathway. This kind of evaluation of existing Arabidopsis methods is necessary as Camelina is a crop that can be used not only as an oil producing plant, but also as a potential plant-based factory for compounds with agronomical, pharmaceutical, or research purposes [24,25]. 

## Figures and Tables

**Table 1:** qRT-PCR Primer list.

*GES*	LP	GACGATTTGGGTACTGCTAAGG
	RP	CTATTCCCTCCCTTCCTTCACT
*G80*	LP	AATCGGCAGAGGAAAAACAATA
	RP	GCGAGGAATTAAGAAGGGAACT
*ACT2*	LP	AAGAGCAGCTCTTCAGTTGA
	RP	AACCTCAGGACAACGGAATC

## Data Availability

Not applicable.

## Supplementary Materials

The following supporting information can be downloaded at: https://www.mdpi.com/article/10.3390/cells11071068/s1, File S1: Supplementary Materials and Methods.

## Abbreviations

Camelina	*Camelina sativa*
Arabidopsis	*Arabidopsis thaliana*
DsRed	*Discosoma* sp. red protein
Kan	Kanamycin
Hyg	Hygromycin
BASTA	phosphinothricin herbicide
qRT-PCR	quantitative real time polymerase chain reaction
TAG	triacylglycerol
cDNA	complementary DNA
