# Supplementary material for: Evaluation of Antibiotic-Based Selection Methods for Camelina sativa Stable Transformants
Source: Cells. 2022 Mar 22;11(7):1068. doi: 10.3390/cells11071068 (PMC8997383; doi:10.3390/cells11071068)
Supplement: Supplementary file 1 [file cells-11-01068-s001.zip › cells-1511302-supplementary.pdf]

Supplementary Materials and Methods

Detailed procedures

In vitro Camelina selection

1. Murashige and Skoog (MS) medium for plates is prepared with 4.3 g/L MS basal salts powder, 0.5 g/L MES, pH 5.7 adjusted with KOH, 8 g agar, 20 g/L sucrose, and 1 mL/L of Gamborg B5 vitamins. Autoclave and cool-down to ~50°C.
2. Transfer 30 mL of MS media to 50 mL Falcon tube and add the proper antibiotic at the desired concentration.
3. In a laminar flow hood, pour the MS media with antibiotic to petri dish and wait until solid to close the lid.
4. Liquid sterilization of Camelina seeds (~200 seeds/15 mL Falcon tube): Add 20% bleach and vortex for 2 min; incubate for 5 min and pour out as much as possible. Repeat these steps 2 more times. Under the sterile laminar flow hood, rinse 3 times with sterile MQ water vortexing, and finally keeping the seeds in water.
5. Transfer seeds to sterile empty petri dish and use sterile tweezers to transfer to MS media plate (50 seeds/plate).
6. Seal the plates with micropore tape and place 48 h at 4°C for stratification.
7. Place the plates in standard long day plant growth conditions (16 h light/8 h dark, approximately 120 µmol photons $m^{-2}s^{-1}$).
8. Grow plates for 20 days.

Positive transformant selection with hygromycin and BASTA.

1. *Camelina sativa* are transformed using standard floral dipping method for camelina [6]. The plasmids used were pH2GW7 and pB2GW7 [8].
2. After drying, seeds are collected and aliquoted for sterilization.
3. Seeds are spread as much as possible in petri dishes (1 per construct) inside a desiccator for gas sterilization.
4. Gas sterilization solution is prepared as follows in a flask: 40 mL MQ water, 6.25 mL bleach and 1.75 mL HCl.
5. Place the solution and the petri dishes inside the desiccator and leave closed overnight for sterilization.
6. Prepare MS media plates with hygromycin 22.5 mg/L and BASTA 15 mg/L.
7. For T1 seeds: Sprinkle as many seeds as possible on MS media plates. For this generation, square 10x10cm plates are recommended.
8. For T2 seeds and on: Plate the seeds with sterile tweezers in MS media plates (50 seeds/plate). Seal with micropore tape and leave 48 h at -20°C for stratification.
9. Place the plates in standard long day plant growth conditions (16 h light/8 h dark, approximately 120 µmol photons $m^{-2}s^{-1}$).
10. Grow plates for 20 days.
